# Volar Perilunate Dislocation: A Case Report and Review of the Literature

**DOI:** 10.2174/1874325000802010057

**Published:** 2008-04-11

**Authors:** B Youssef, S.C Deshmukh

**Affiliations:** The Hand Service, The Royal Orthopaedic Hospital, Northfield, Birmingham, UK

## Abstract

Perilunate dislocations, lunate dislocations and perilunate fracture dislocations are rare injuries comprising of less than 10% of all wrist injuries. Volar peri-lunate dislocations (VPLDs) account for less than 3% of perilunate dislocations. These severe carpal injuries occur after high-energy trauma to the wrist and falls on the outstretched hand. We present a case of a missed VPLD who developed parasthesia in the distribution of the median nerve 18 months after the initial injury. A plain film radiograph revealed a stage II VPLD. Nerve conduction studies confirmed compression of the median nerve at the carpal tunnel. VPLDs are extremely rare injuries. A quarter of perilunate dislocations are missed on initial presentation. The outcome is poor for missed injuries and this patient is aware that a wrist fusion may be required in the future for to treat symptoms.

## INTRODUCTION

A 53 year-old unemployed right hand dominant gentleman presented with numbness in his left hand. He fell onto his hyperextended wrist 18 months ago. He did not seek any medical attention at the time because it did not cause any significant symptoms. Only when the parasthesia became permanent did he visit his general practitioner and was subsequently referred to the regional hand service.

His past medical history included type II diabetes, cervical spondylosis, dorsal lumbar scoliosis and osteoarthritis of both knees. On examination there was thickening of the carpus and a slight prominence of the lunate on the dorsum of the hand. Wrist dorsiflexion was 70 degrees, volarflexion 45 degrees, radial deviation 10 degrees and ulnar deviation 20 degrees. A full range of pronation and supination was possible. Sensation was reduced in the distribution of the median nerve. No thenar, hypothenar or intrinsic muscle wasting was present. Grip strength was 36 kg in his right hand and 25 kg in the left hand. Key pinch was 8 kg bilaterally. The Disability of Arm Shoulder and Hand (DASH) score was 68 points. This outcome measure is a 30-item, self-reported questionnaire designed to measure physical function and symptoms in people with musculoskeletal disorders of the upper limb. It is a reliable instrument that can be used to assess any or all joints in the upper limb. Compression of the median nerve was a result of the volar displacement of the carpus compressing the nerve and this was confirmed detected with nerve conduction studies.

**Fig. (1):**
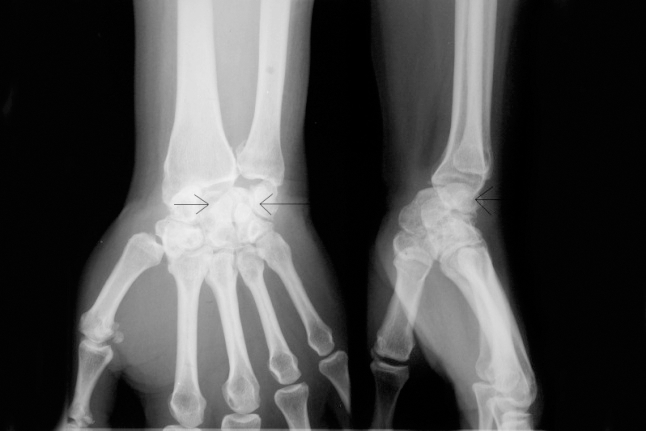
The arrows identify the lunate and the relative volar dislocation of the remaining carpus can be seen.

A plain film radiograph revealed a stage II volar perilunate dislocation (VPLD) and radio-scaphoid osteoarthritis (Fig. 1). This is a very rare injury and to our knowledge a presentation of VPLD this length of time after injury has not been previously reported in the literature. He currently remains on the waiting list for a carpal tunnel decompression.

## DISCUSSION

Perilunate dislocations and fracture dislocations are rare injuries [1]. A considerable proportion of them are frequently missed. 41 of 166 patients sustaining these injuries reviewed in a multicenter study were missed on initial presentation [2]. VPLD are extremely rare only accounting for 3% of all perilunate dislocations. For pure dislocations the primary injury is ligamentous, classically disrupting the scapho-lunate, capito-lunate and lunato-triquetral joints [3].

It is not possible to illicit the mechanism of injury from the plain film radiographs, however theories have been put forward based on the patient history and on cadeveric studies. Two mechanisms of VPLD have been described in the literature. The first was described in 1960 by Aitken and Nalebuff; they proposed that a force directed to a palmar flexed wrist forces the capitate anteriorly, resulting in a volar translation of the carpus [4]. Further descriptions by Pournas and Kappas in 1979 [5], Chamay in 1981 [6] and Carmicheal in 2005 [7] supported this theory.

Green and O’ Brien described VPLDs in two patients. One patient’s injury was an isolated VPLD and the other involved a fracture of the scaphoid. They believed that a fall onto a hyper-extended wrist with the forearm supinated resulted in a pure dislocation, and a fall onto a hyperextended wrist without forearm supination caused a fracture dislocation [8]. They elucidated this information from a dissected carpus of one of the patients who had died as a result of other injuries.

A cadaveric study performed by Niazi in 1996 [9] supported the Green and O’Brien theory. He described three elements to the injury:

- Rupture of the volar ligaments.
- Supination of the proximal segment rupturing the scaphotrapezial ligament.
- A significant momentum of force causing posterior shearing.

A trans-scaphoid VPLD in association with a radial styloid fracture has been reported in a skeletally immature patient who fell onto the dorsum of his hand with the wrist palmarflexed [6].

In Herzberg’s series of perilunate dislocations, 23% presented with acute carpal tunnel syndrome and 33% of those with missed diagnosis went on to present with carpal tunnel syndrome [2]. No comment was made on the outcome of carpal tunnel decompression in this group of patients.

Management of pure dislocations includes closed reduction and percutaneous fixation for 8 weeks. Trans-scaphoid fracture dislocations are treated with open reduction and internal fixation of the scaphoid through a volar approach [8]. In the multi-centre review of perilunate dislocations good results were achieved by operative fixation of associated scaphoid fractures, whereas one case treated with closed reduction and plaster immobilisation went onto scaphoid non-union and subsequent carpal instability [2]. The outcome was significantly worse for those patients who underwent surgical treatment much later after the initial injury.

## CONCLUSION

VPLDs are extremely rare injuries. Two mechanisms have been described in the literature: a dorsally applied pressure to a palmar-flexed wrist or forced hyperextension of the wrist. A substantial force is usually required to cause perilunate dislocations, such as falls from a height or road traffic collisions. This case report supports the Green and O’Brien theory of falling onto a hyperextended wrist. Management of acute injuries involves closed reduction and percutaneous fixation for pure dislocations or open reduction and internal fixation for dislocations involving fractures.

The delayed presentation of this VPLD has dictated the choice of conservative management for the dislocation. His median nerve compression symptoms can be adequately managed with a straight forward carpal tunnel decompression. The patient is aware that a wrist fusion may be necessary in the future if he becomes symptomatic from the osteoarthritic changes.
